# Peripartum women’s perspectives on research study participation in the OneFlorida Clinical Research Consortium during COVID-19 pandemic

**DOI:** 10.1017/cts.2022.476

**Published:** 2022-10-10

**Authors:** Ke Xu, Chu J. Hsiao, Hailey Ballard, Nisha Chachad, Callie F. Reeder, Elizabeth A. Shenkman, Elizabeth Flood-Grady, Adetola F. Louis-Jacques, Erica L. Smith, Lindsay A. Thompson, Janice Krieger, Magda Francois, Dominick J. Lemas

**Affiliations:** 1 Department of Health Outcomes and Biomedical Informatics, University of Florida College of Medicine, Gainesville, FL, USA; 2 Department of Anthropology, University of Florida, Gainesville, FL, USA; 3 Department of Obstetrics & Gynecology, University of Florida College of Medicine, Gainesville, FL, USA; 4 STEM Translational Communication Center, College of Journalism and Communications, University of Florida, Gainesville, FL, USA; 5 Department of Pediatrics, University of Florida College of Medicine, Gainesville, FL, USA

**Keywords:** Clinical research recruitment, pregnancy, breastfeeding, barriers, research participation

## Abstract

**Introduction:**

The COVID-19 pandemic created an unprecedented need for population-level clinical trials focused on the discovery of life-saving therapies and treatments. However, there is limited information on perception of research participation among perinatal populations, a population of particular interest during the pandemic.

**Methods:**

Eligible respondents were 18 years or older, were currently pregnant or had an infant (≤12 months old), and lived in Florida within 50 miles of sites participating in the OneFlorida Clinical Research Consortium. Respondents were recruited via Qualtrics panels between April and September 2020. Respondents completed survey items about barriers and facilitators to participation and answered sociodemographic questions.

**Results:**

Of 533 respondents, most were between 25 and 34 years of age (*n* = 259, 49%) and identified as White (*n* = 303, 47%) and non-Hispanic (*n* = 344, 65%). Facebook was the most popular social media platform among our respondents. The most common barriers to research participation included poor explanation of study goals, discomforts to the infant, and time commitment. Recruitment through healthcare providers was perceived as the best way to learn about clinical research studies. When considering research participation, "myself" had the greatest influence, followed by familial ties. Noninvasive biological samples were highly acceptable. Hispanics had higher positive perspectives on willingness to participate in a randomized study (*p* = 0.009). Education (*p* = 0.007) had significant effects on willingness to release personal health information.

**Conclusion:**

When recruiting women during the pregnancy and postpartum periods for perinatal studies, investigators should consider protocols that account for common barriers and preferred study information sources. Social media-based recruitment is worthy of adoption.

## Introduction

Clinical trials involving maternal and infant populations are crucial to improving perinatal health outcomes. The COVID-19 pandemic created an unprecedented need for population-level clinical trials focused on discovery of life-saving therapies [1]. Despite these observations, US clinical trial registry data demonstrate that most clinical trials of COVID-19 therapies have excluded pregnant women [2]. As a result, there is limited data on the safety, effectiveness, and fetal risk evaluation for COVID-19 treatment and drugs for pregnant individuals and healthcare providers [3]. This is not an isolated example as pregnant women have been systematically excluded from medical treatments that have few to no known safety issues during pregnancy [4]. Compounding the problem are data showing that women who lack access to healthcare or do not seek prenatal care are often excluded from research opportunities [5], leading to population underrepresentation of low resource and hard-to-reach individuals. Collectively, these data demonstrate that exclusion of pregnant individuals from clinical trials contributes to research results that have limited generalizability to perinatal populations and potential biases that may impact health outcomes [6,7].

Previous work in perinatal research participation has identified facilitators and barriers to research participation across individual [8–10], family [11], and community factors [10,12]. Individual factors that facilitated research participation included cash or gift incentives [9], minimizing the effort required of women to participate [8], and participants’ motivations [10]. Family-level factors include partners’ opinion [11] and direct benefit to the family unit [13]. Facilitators at the community level included lactation support [10], healthcare provider influences [12], and the trust between participants and providers [12]. Barriers to research participation at individual level included concerns about privacy and confidentiality as well as time commitment [11,12,14]. Community-level barriers included inadequate clinical support [15], study risks [16], and the uncertainty around characterizing the research or intervention [12]. However, these observations were all conducted before the COVID-19 pandemic, and there is a critical need to understand the perspectives of these populations during the COVID-19 pandemic. There is currently limited information on the perception of research participation held by perinatal populations during the COVID-19 pandemic.

In this study, we collected questionnaires between August 2020 and September 2020 during the first wave of the pandemic in Florida. Florida is the fourth ranking US state in terms of number of births [17]. The primary objective of this study was to evaluate research participation experiences and preferences among Florida women who were eligible to participate in clinical research studies related to pregnancy and postpartum during the COVID-19 pandemic. Ultimately, our goal was to improve the success rate of research recruitment of Florida women who are eligible to participate in clinical research studies.

## Materials and Methods

### Respondents and Recruitment

The OneFlorida Clinical Research Consortium is a statewide, collaborative partnership comprised of 11 health systems, numerous providers, and insurers across the state of Florida [18]. It includes 17.2 million individuals who received healthcare in Florida between January 2012 and the present [19]. The infrastructure of OneFlorida is increasingly used to incorporate data from numerous sites for clinical data analysis [20,21]. Because the Oneflorida Clinical Research Consortium has real-life data, understanding the facilitators and barriers to clinical research participation experienced by peripartum women living within the catchment areas of OneFlorida sites is important. The OneFlorida sites selected for inclusion included Tallahassee, Jacksonville, Gainesville, Orlando, Tampa, and Miami. Eligible respondents were female, aged 18 years or older, currently pregnant or had an infant less than or equal to 12 months, and lived in Florida within 50 miles of sites participating in the OneFlorida Clinical Research Consortium. Qualtrics, a commercial survey sampling and administration company, was contracted to recruit respondents and implement the survey. Qualtrics actively maintains channels of recruitment and research panels designed to be demographically representative of the overall population [22]. Prospective respondents (female aged 18+ years living in qualifying zip code) received an email invitation to complete prescreening questions. These questions verified female sex, age 18+ years, residence in a qualifying zip code, and status of being currently pregnant or having an infant 12 months old. Respondents from Qualtrics’ panels likely to meet screening criteria were invited to participate. Additionally, Qualtrics’ sample partners randomly selected respondents for surveys for which respondents were likely to qualify. Respondents who met eligibility criteria consented to taking a one-time, 30-minute online Qualtrics survey. Approval by the University of Florida’s Institutional Review Board was obtained on 2018/8/13 (#201801273).

### Questionnaires

**Fig. 1.**
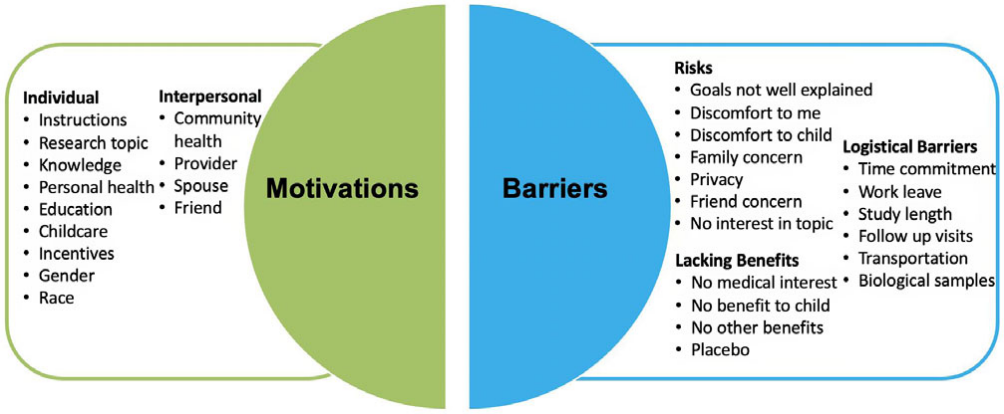
Pie chart for barriers and facilitators to clinical research participation. The factors considered as facilitators are individual factors and interpersonal factors. The factors considered in barriers are risks, logistical barriers, and lacking benefits.

**Fig. 2.**
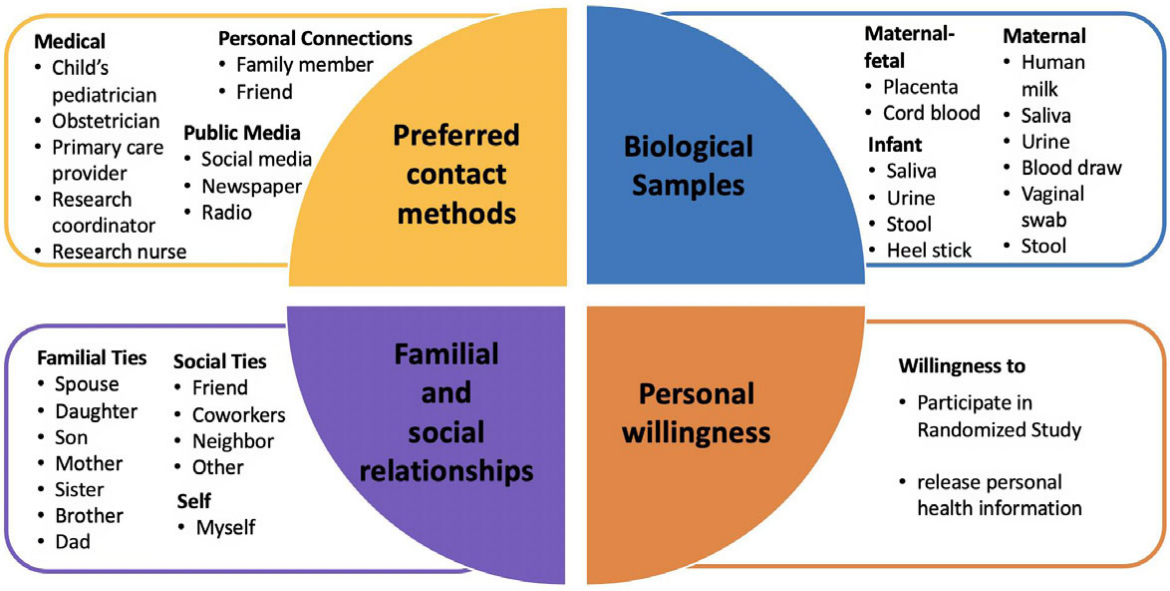
Pie chart for influencing factors considered in clinical research preferences. They are 1) Preferred contact methods for information about a clinical research study, 2) Personal willingness, 3) Biological samples, and 4) Familial and social relationships.

Respondents completed a survey with items that included sociodemographic factors, prior experiences with clinical research, and social networks utilization (see survey instrument in Supplementary Information 1). We also asked respondents’ motivators/barriers to clinical research participation (Fig. 1), preferred source of research study information, comfort with biological sample collection, willingness to release electronic health record (EHR), and comfort with study randomization (Fig. 2). Questionnaires were constructed through a combination of previously published scales and questions (e.g. sociodemographic questions) as well as measures adapted from existing items designed to collect information about perceptions and experiences with clinical research (e.g. from the Health Information National Trends Survey (HINTS) [23] and based on findings from formative research our group conducted with women who were pregnant and breastfeeding as part of the larger pilot study [10]). Sociodemographic questions included perinatal status, maternal age, gravidity, education, race and ethnicity, household income, health insurance, Women Infants and Children (WIC) eligibility, relationship status, and breastfeeding status. Responses were reported as five-point Likert scales. The Likert scale for preferred source of information about clinical research studies, importance of social connections related to clinical research participation, and motivators and barriers to research participant was coded 1 (strongly disagree) through 5 (strongly agree). The scale for comfort in providing biological sample collections ranged from 1 (very uncomfortable) to 5 (very comfortable). Willingness to release personal health information and participate in a randomized study were coded 1 (very unlikely) through 5 (very likely).

### Data Analysis

Study data were managed using REDCap electronic data capture tools [24,25]. Data were entered and validated by separate team members to ensure accuracy. Participant willingness to release personal health information and willingness to participate in a randomized study were also analyzed by education level and race, ethnicity, and education. We used R [26] (version 4.0.5) to analyze all data. Descriptive statistics were calculated using gtsummary[27] (version 1.4.1). Responses to check-all-that-apply questions were visualized using HH [28] (version 3.1.43) and lattice [29] (version 0.20.44). Histograms for comparison by race/ethnicity and education were constructed using ggplot2 [30] (version 3.3.3). Pearson’s Chi-square tests were performed to examine the effect of race, ethnicity, and education on positive (very likely and somewhat likely) vs. negative (very unlikely and somewhat unlikely) willingness to release personal health information and willingness to participate in a randomized study.

## Results

### Recruiting

A total of 2984 invitations were sent out, with 1705 respondents meeting the prescreening questions (female, age 18+ years, resided in zip code of interest) and starting the survey. Of the 1705 respondents, 1462 consented and completed the survey (114 did not proceed past the consent question; 14 did not complete the survey; 115 answered prescreening questions after sample parameters were met), for a response rate of 85.7%. An additional 929 respondents were excluded for the following reasons: 22 indicated an age younger than 18 years on the survey, 17 were suspicious for low-quality data (e.g. survey completion under five minutes), and 890 had an IP address outside Florida state. Ultimately, 533 respondents were included in this study.

### Respondent Characteristics

Respondent characteristics were described in Table 1. Of the 533 respondents, 55.0% were mothers of an infant, 42.4% were pregnant, and 5.6% satisfied both categories. Whites (47.3%) accounted for a larger proportion of respondents compared to patients in OneFlorida (45.9%). However, this proportion of respondents is more similar than the proportion of Hispanic respondents (35.5%) compared to Hispanic patients in OneFlorida (23%). A high proportion of respondents held a college degree or higher (70.7%). The majority of respondents were between 18 and 34 years of age (81.4%). More than 60% of respondents had a household income of $37,000 or more and almost 82% of respondents had Medicaid or private insurance. Nearly, a third of respondents were previously asked to participate in a clinical research study, but less than a quarter of individuals actually participated in the research. Of those with prior experience in a research study, 57.4% were in studies related to pregnancy. The most common research topic they participated in was allergy and immunology, closely followed by pain relief and diabetes (Supplementary Table 1). Respondents were interested in social media utilization and pregnancy-related apps (Supplementary Table 2). The majority of respondents used an Apple smart phone (68.9%), followed by Samsung (17.1%) to receive study information. The most commonly used social media apps were Facebook (43.3%) and Instagram (31.7%), with 71.5% using pregnancy-related apps and 45.4% using breastfeeding-related apps.

**Table 1.** Participant characteristics

Characteristic	Frequency (*n*)	Percent (%)
**Perinatal Status**		
Pregnant	226	42.4
Mother	277	55.0
Pregnant and Mother	30	5.6
**Recruitment Method**		
Other	217	40.7
Social media	194	36.4
Email	65	12.2
Word of mouth	41	7.7
Phone call	16	3.0
**Maternal Age**		
18–24 years	175	32.8
25–34 years	259	48.6
35–44 years	87	16.3
45+ years	12	2.3
**Gravidity**		
0	103	19.3
1	152	28.5
2	115	21.6
3+	165	31.6
**Maternal Education**		
Graduate degree	124	23.4
College degree	250	47.3
High school degree	122	23.1
8th grade or less	5	0.9
Technical/vocational degree	28	5.3
**Maternal Race**		
White	303	56.8
Black	105	19.7
Asian	24	4.5
Multiple	70	13.1
Other	31	5.8
**Maternal Ethnicity**		
Not Hispanic	344	64.5
Hispanic	189	35.5
**Household Income**		
$0–$37,000	204	39.2
$37,001-–$75,000	213	40.9
$75,000 or higher	104	20.0
**Health Insurance**		
Medicaid	231	44.3
Private	194	37.2
Other	42	8.0
Military	29	5.6
No insurance	26	5.0
**WIC Eligibility**		
Yes	311	65.5
No	164	34.5
**Relationship Status**		
Engaged or married	312	58.5
In a committed relationship	143	26.8
Single	64	12.0
Separated or divorced	9	1.7
Widowed	5	0.9
**Breastfeeding Status**		
Yes	191	62.2
No	116	37.8

### Motivators and Barriers to Research Participation

Respondents were highly motivated to participate in research if the study had well-explained instructions or benefited the health of the community (Supplementary Figure 1). Respondents agreed with the following facilitators of participation: knowledge gained from my participation can benefit someone else in the future; my participating in research largely dependents on whether the study offers educational benefits and support groups; this research topic is interesting for me; and, the fact that the findings benefit my own health plays an important role in my research participation. The gender and race of the researcher team conducting the study were the least important factors motivating research participation. The largest barriers to research participation were ambiguity in the research study goals, discomforts to the child, family concern, time commitment, lack of support from medical/clinical staff, and lack of interest in research from medical staff (Supplementary Figure 2). The need to provide biological samples or a placebo design, discomfort to respondents, and receiving the placebo treatment in clinical research were identified as least likely to be considered barriers to research participation.

### Clinical Research Preferences

**Fig. 3.**
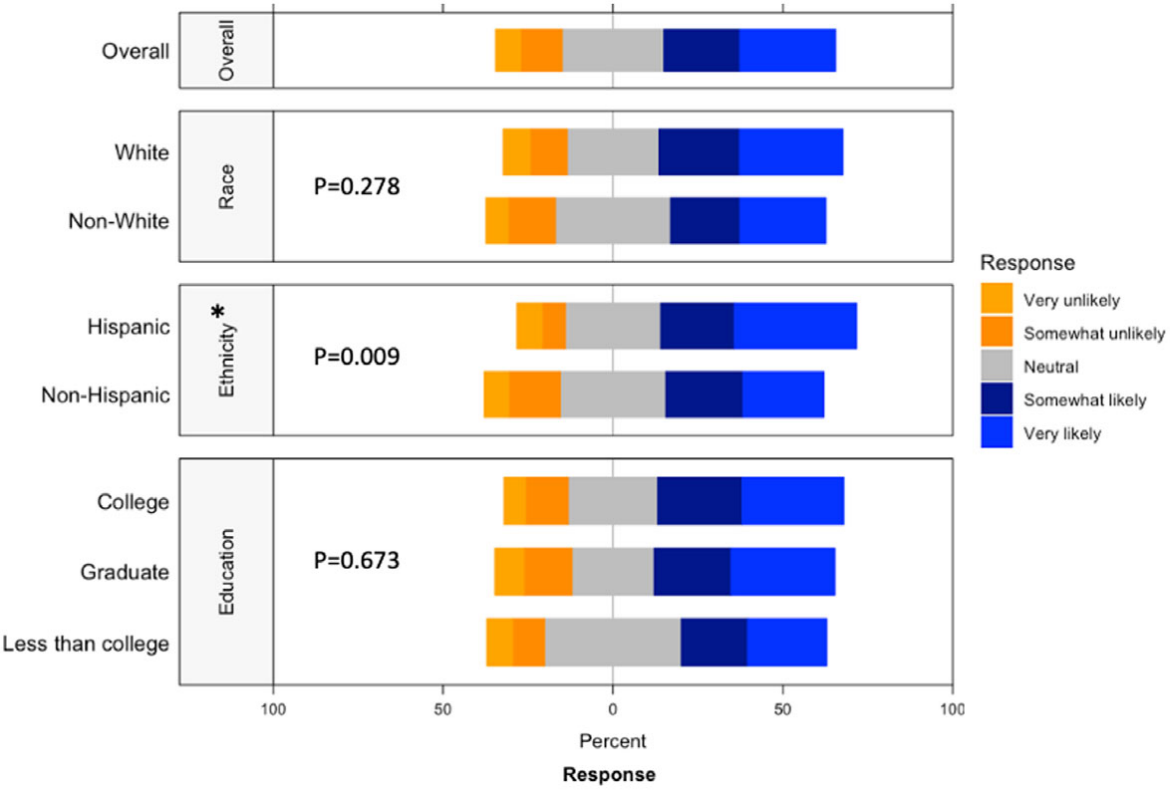
Willingness to participate in a randomized study by race, ethnicity, and education. This graph shows the Likert scale of willingness to participate in a randomized study. The graph visualizes the proportion of the responses reporting very unlikely, unlikely, neutral, likely, and very likely towards this question by race, ethnicity and education. Gray stands for neutral, blue stands for positive attitude, and orange stands for negative attitude.

Respondents strongly preferred to receive information on actively recruiting research studies from their healthcare provider, whether it was the child’s pediatrician, their obstetrician, or a primary care provider (Supplementary Figure 3). While the respondent’s own opinions were deemed most important in making the decision to participate, the opinions of familial ties (e.g. spouse, children, parents, and siblings) were more important than other social ties (e.g. friend, coworker, neighbor; Supplementary Figure 4). We found that female connections were consistently ranked higher than male connections (i.e. mother ranked higher than father, daughter ranked higher than son, and sister ranked higher than brother). When asked about willingness to participate in a randomized study, ethnicity (*p* = 0.009) had a significantly higher positive response while race (*p* = 0.278) or education (*p* = 0.673) had no significant difference in perspective (Fig. 3). Compared to non-Hispanics, Hispanics had higher positive perspectives on willingness to participate in a randomized study.

**Fig. 4.**
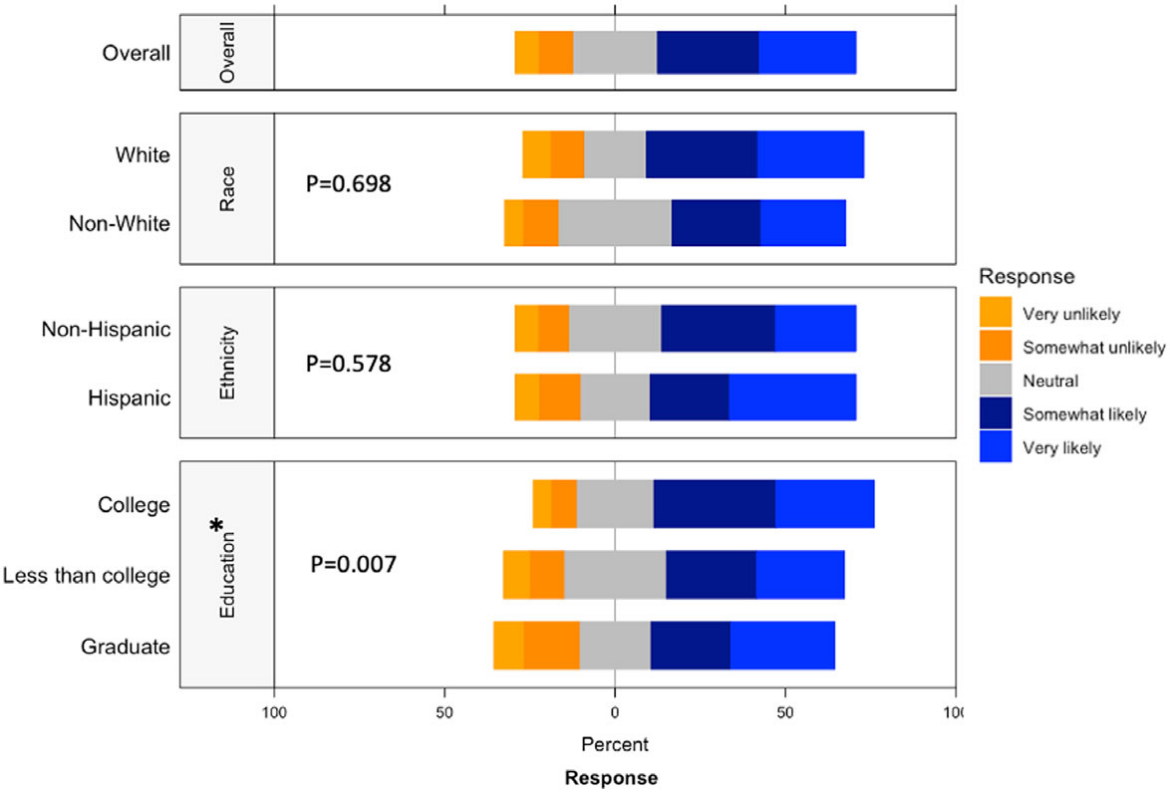
Willingness to release personal health information by race, ethnicity, and education. This graph shows the Likert scale of willingness to release personal health information by race and education. The graph visualizes the proportion of the responses reporting very unlikely, unlikely, neutral, likely, and very likely towards this question by race, ethnicity and education. Gray stands for neutral, blue stands for positive attitude, and orange stands for negative attitude.

Respondents were most comfortable providing noninvasive biological samples (i.e. human milk and maternal saliva) to researchers (Supplementary Figure 5). For maternal samples, respondents were least comfortable providing vaginal swabs and stool samples. For infant samples, respondents were least comfortable with research studies requiring infant heel sticks, a discomfort on par with providing placenta or cord blood tissues. The majority of respondents were either somewhat likely or very likely to release personal health information to researchers, whereas about a quarter of respondents had a neutral stance (Fig. 4). Of the three factors analyzed, race (*p* = 0.698) and ethnicity (*p* = 0.578) showed no effect on perspectives of willingness to release personal health information, while education (*p* = 0.007) showed a statistically significant effect.

## Discussion

### Overview

There is currently limited information on the perceptions of research participation among perinatal populations, a population of particular interest during the COVID-19 pandemic due to the emergence of many clinical questions (i.e. questions about vertical transmission). In comparison to current literature on barriers/facilitators to research participation, this study also surveyed respondent’s use of social media and comfort with different types of biological specimen collection. Social media platforms, as well as pregnancy and breastfeeding-related apps, were widely used among respondents. Recruitment through social media platforms or existing pregnancy-related apps may be a potential recruitment method, particularly during a pandemic when more traditional face-to-face recruitment methods are limited. Respondents highlighted the importance of understanding the benefits of the study and preferred well-established protocols related to noninvasive biological samples collection. More than half of respondents responded positively to providing personal health information and participating in randomized studies.

### Social Media

As COVID-19 continues to influence the way clinical activities are conducted, there may be fewer face-to-face, clinician-patient opportunities for participant recruitment [31]. The social media advertising campaign is an effective and efficient strategy to post recruitment and study information to collect large-scale, nationwide data on COVID-19 within a short time period [32]. Facebook was the most popular social media platform among our respondents. Benefits of recruitment through Facebook include added racial and ethnic diversity to the participant pool [33,34]. Our recruitment through Qualtrics included social media ads on Facebook. As such, our respondent pool was 56.8% White in this study compared to 69.0% White in a prior study that recruited solely from face-to-face encounters in an academic hospital setting [14]. Social media-based recruitment may be a particularly beneficial strategy to diversify the clinical research recruitment arsenal as well as diversify the participant pool to be more representative of the socioeconomic and racial/ethnic makeup of the population.

### Motivations and Barriers

Motivations and barriers largely influence research participation decision-making [3]. We found that the most popular source to learn about recruiting clinical studies was through healthcare providers, even during a pandemic that has been documented to have magnified mistrust in the biomedical research complex [35]. *Frew et al*. concluded that the respect afforded prenatal providers can overcome recruitment difficulties for women during pregnancy [9]. Our finding that significant barriers to participation were poor explanation of study goals, the physical discomfort of participation, and time commitment were consistent with existing studies [12]. As such, it would appear that the COVID-19 pandemic did not drastically change the motivations and barriers of peripartum women to participate in clinical research studies.

### Biological Specimen Collection

Overall, respondents were more receptive to providing noninvasive samples (e.g. human milk) than invasive samples (e.g. maternal vaginal swab) that cause more discomfort for research purposes. Receptiveness to providing a particular tissue type may also be influenced by respondents’ knowledge of why and how such samples are used. One study found that parents’ information on cord blood use in research was varied, fragmented, and inconsistent [36]. The concerns with providing samples may differ by tissue type. *Bailey et al.* reported barriers to stool collection included physical impediments such as constipation, embarrassment, and unfamiliarity due to stool collection not being a routine clinical procedure [37]. Similarly, informative interviews conducted by Lemas and colleagues identified stool collection as a barrier to research participation in a cohort of 40 pregnant and breastfeeding individuals [10]. Although respondents in our study highlighted stool collection as a barrier to research participation, we also extend these findings of *Lemas et al.* by showing that biospecimen collection of human milk, saliva, and urine were more acceptable than stool collections [10]. Collectively, our results highlighted the need for general education around biospecimen collection of stool, cord blood, and placental tissues to improve participant comfort in donating such tissues to research.

## Strengths and Limitations

Our study has several strengths. First, our study recruited more women who identified as being from a minority racial or ethnic group than US Census [38] estimates suggest is currently representative of Florida state. It is important to note that this patient population is generally underrepresented in clinical research studies. Our analysis therefore is able to bring to light data from minorities that have historically been and still are excluded from research. Second, responses were collected from those living within the catchment area of the OneFlorida Clinical Research Consortium.

Compared to traditional recruiting methods that largely depend on healthcare provider referrals (a method that selects for those who can access healthcare providers), those recruited to our survey were done primarily through online methods (i.e. email and social media). It is possible that in world still grappling with the pandemic, reduced face-to-face encounters may result in a shift towards more online recruitment methods. As such, our sample better reflect those responsive to online recruitment. Another strength is that the perspectives captured in this paper belong to people who could actually be approached for recruitment into clinical trials at OneFlorida sites. Additionally, as small samples may undermine the internal and external validity of a study [39], another strength of our study is a robust sample size (*n* = 533) compared to other participant recruitment studies of pregnant or lactating women [7,10,12,40].

Some limitations apply to our findings. First, we lack a comparable dataset on perspectives of prepartum women before COVID-19 pandemic. As such we cannot draw conclusions about how the pandemic has impacted views on clinical research participation. However, it is likely that our respondent sample, recruited from largely online methods, may better reflect recruitment in a society with limited ability to recruit traditionally via face-to-face encounters. Recognizably, our online recruitment methods likely introduced some bias into our sample. For example, it is well documented that those with higher education are more likely to complete online surveys than those with lower education [41]. Second, we only focused on peripartum women in Florida instead of nationwide populations and the respondents were much more highly educated than US Census [38] numbers would predict, which limited the generalizability of our findings. Third, we lacked respondents outside our selected sites of OneFlorida Clinical Research Consortium, which may have led to recruitment bias against people residing in more rural areas. Fourth, we were unable to obtain the distribution of demographics of our targeted population, thereby limiting our ability to thoroughly understand the sampling bias in our findings.

## Conclusion

Clinical research involving pregnancy is unique and complex in that the outcomes are relevant not only for the mother but also for the fetus. This study collected data among participants within or near the OneFlorida Clinical Research Consortium, a centralized research patient data repository that contains real-world data in the third largest state in the USA [19]. The OneFlorida Clinical Research Consortium is increasingly used to incorporate EHRs into clinical data analysis for studies ranging from pediatric chart reviews to demographics of adult hypertension [20,21]. Thus, it was important to understand the motivators and facilitators to clinical research of women residing in the catchment areas of OneFlorida sites. Social media apps were commonly used and represented potential for application in today’s clinical trials as recruitment strategies [42], particularly as the pandemic has changed the frequency of face-to-face encounters [32]. Researchers should communicate research information to respondents through medical institutions and healthcare providers. In order to effectively improve the recruitment success rate of perinatal studies, recruiters should focus on protocols that account for physical discomfort, communication mode, and time commitments. Finally, noninvasive biological specimens collection for research studies must be carefully considered, with clear explanations of how those specimens will be used and advance health science.

## Supplementary material

For supplementary material accompanying this paper visit https://doi.org/10.1017/cts.2022.476. click here to view supplementary material
