# Supplementary material for: Peripartum women’s perspectives on research study participation in the OneFlorida Clinical Research Consortium during COVID-19 pandemic
Source: J Clin Transl Sci. 2022 Oct 10;7(1):e24. doi: 10.1017/cts.2022.476 (PMC9879925; doi:10.1017/cts.2022.476)
Supplement: Supplementary file 1 [file ctssup.zip › S2059866122004769sup001.docx]

### **SUPPLEMENTARY MATERIALS**

####


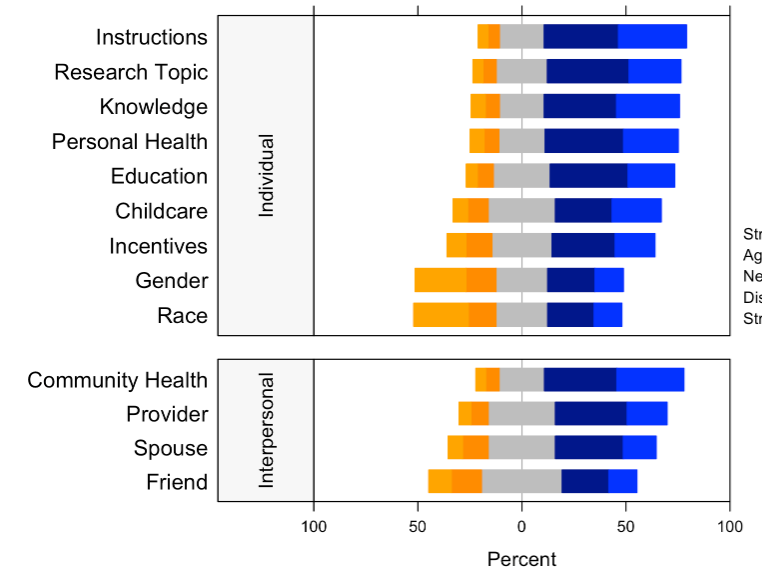

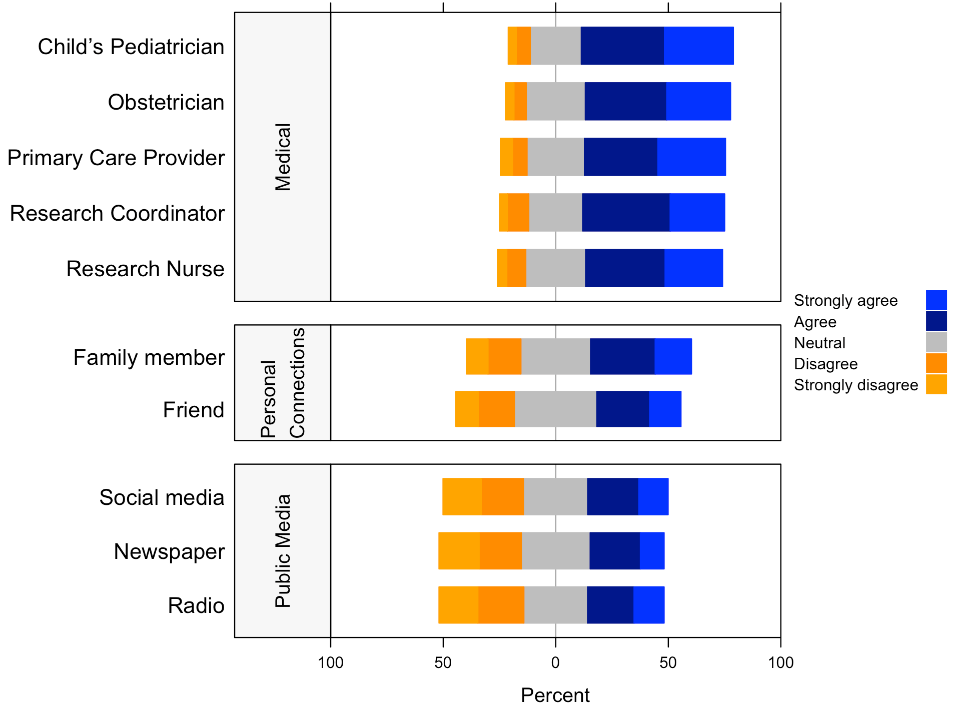

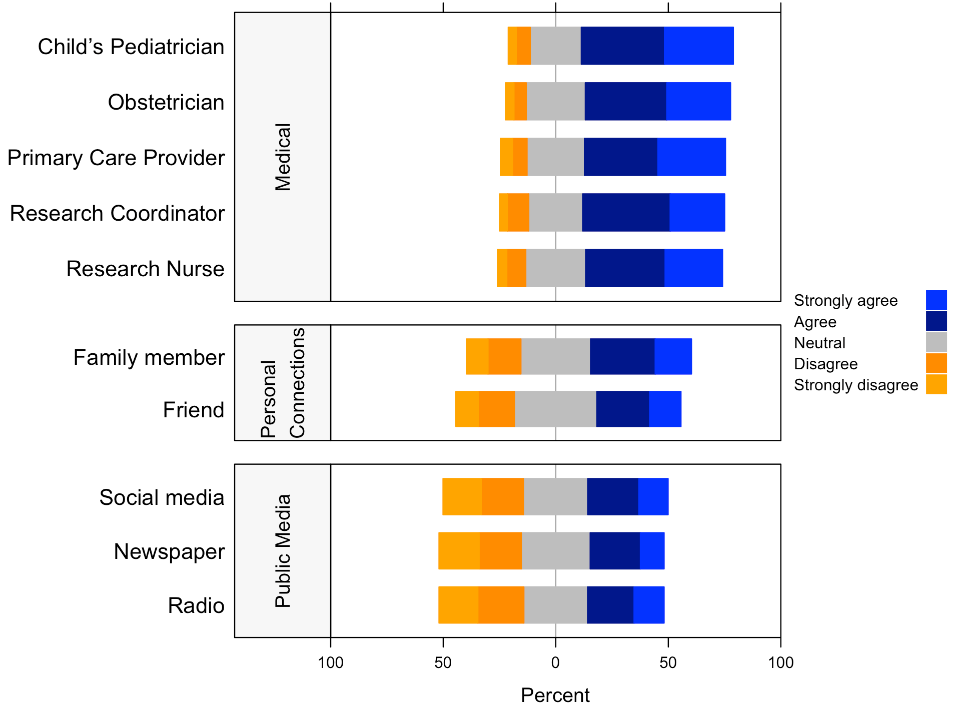

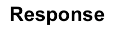


#### **Figure 1. Individual and interpersonal factors motivating research participation.** This graph shows the Likert scale of factors motivating research participation. The graph visualizes the proportion of the responses reporting strongly disagree, disagree, neutral, agree, and strongly agree towards the 13 items of factors motivating research participation. Gray stands for neutral, blue stands for positive attitude, and orange stands for negative attitude.

#### **
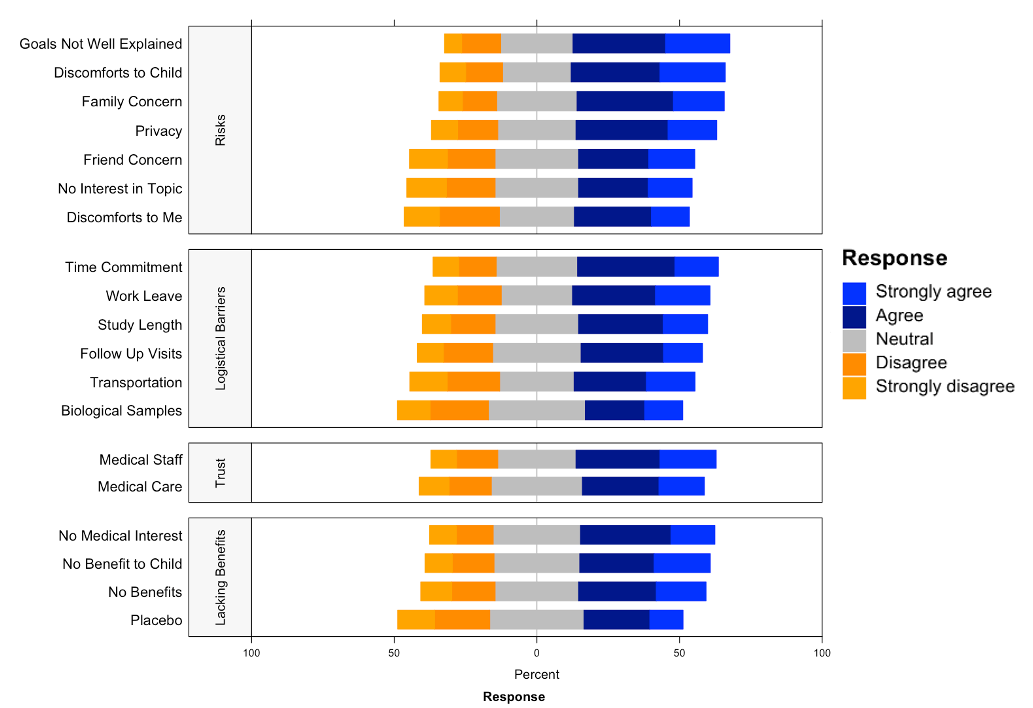
**

#### **Figure 2. Barriers to research participation.** This graph shows the Likert scale of barriers to research participation. The graph visualizes the proportion of the responses reporting strongly disagree, disagree, neutral, agree, and strongly agree towards the 19 items of barriers to research participation. Gray stands for neutral, blue stands for positive attitude, and orange stands for negative attitude.


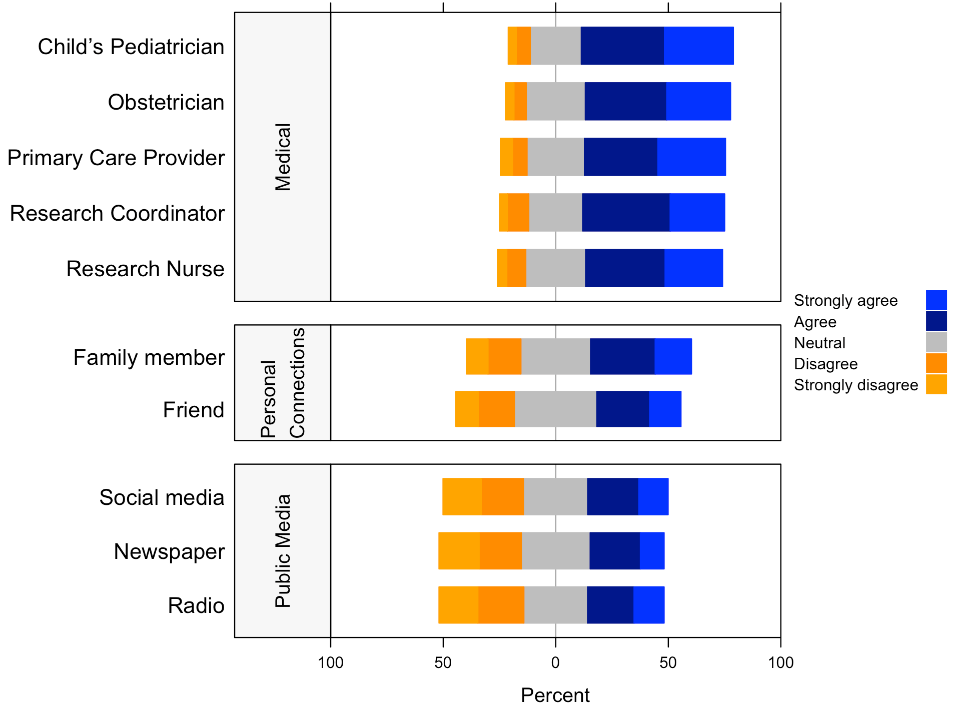

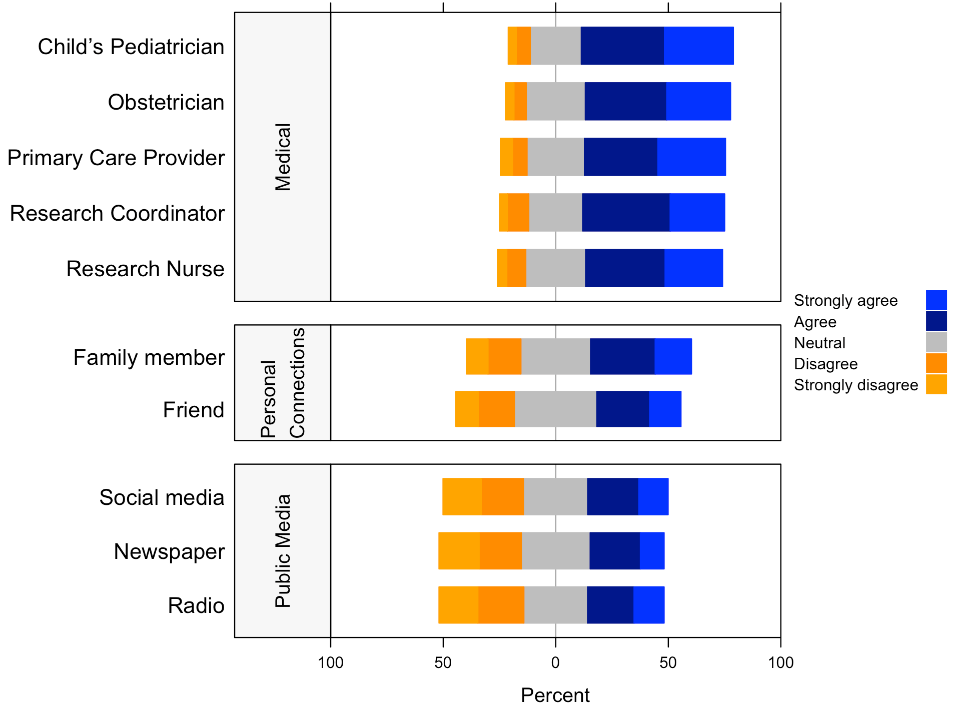

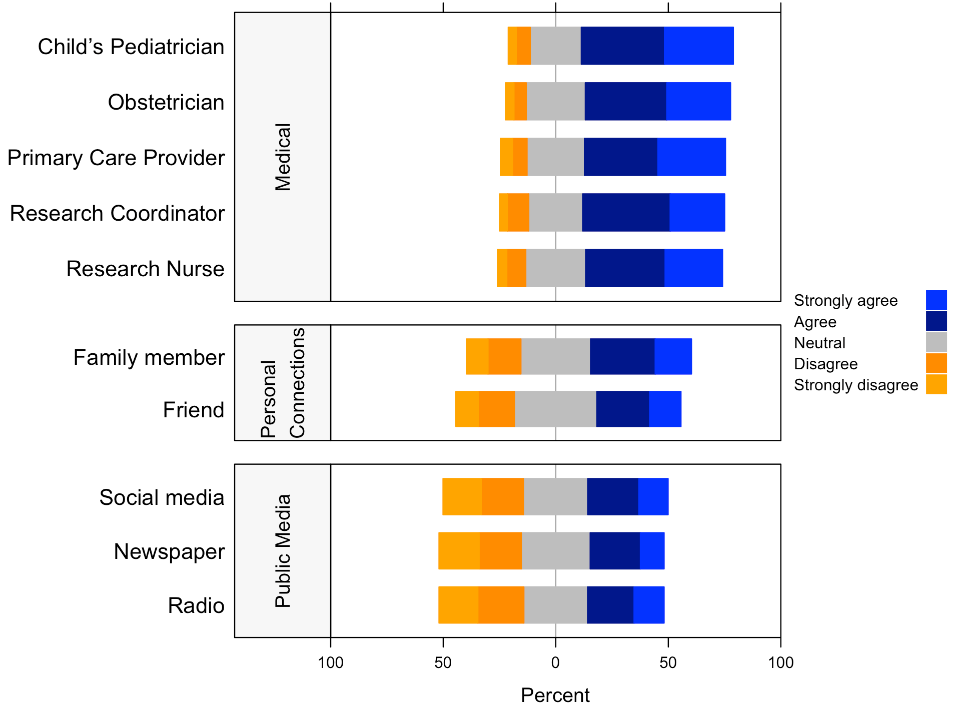

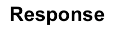


**Figure 3. Preferred contact methods for information about a clinical research study.**  This graph shows the Likert scale of preferred contact methods for information about a clinical research study. The graph visualizes the proportion of the responses reporting strongly disagree, disagree, neutral, agree, and strongly agree towards the 10 items of preferred contact methods for information about a clinical research study. Gray stands for neutral, blue stands for positive attitude, and orange stands for negative attitude.

####


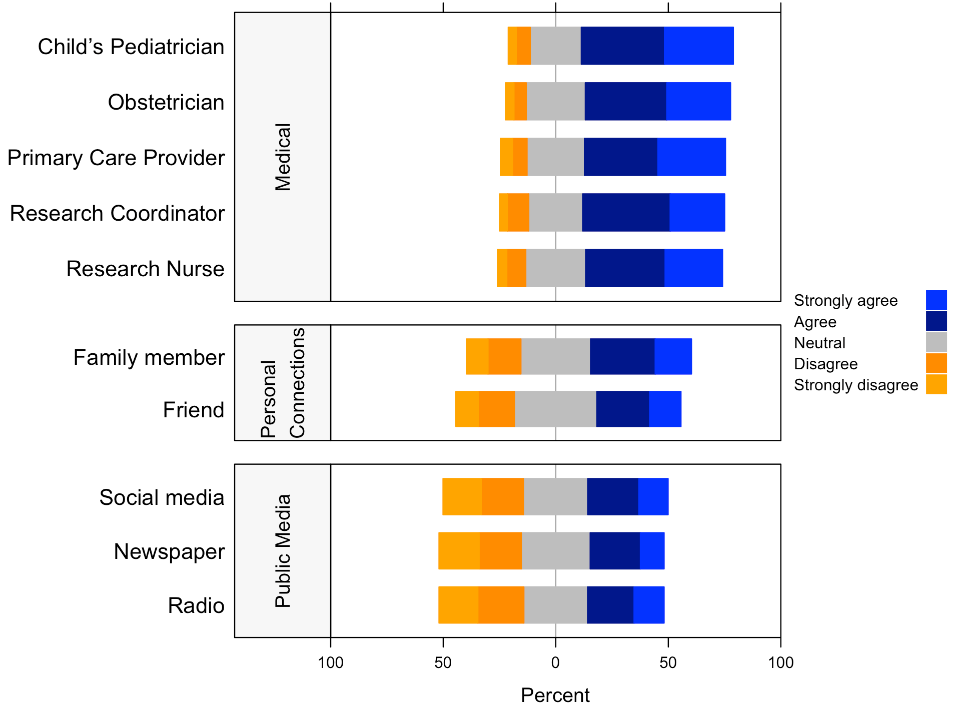

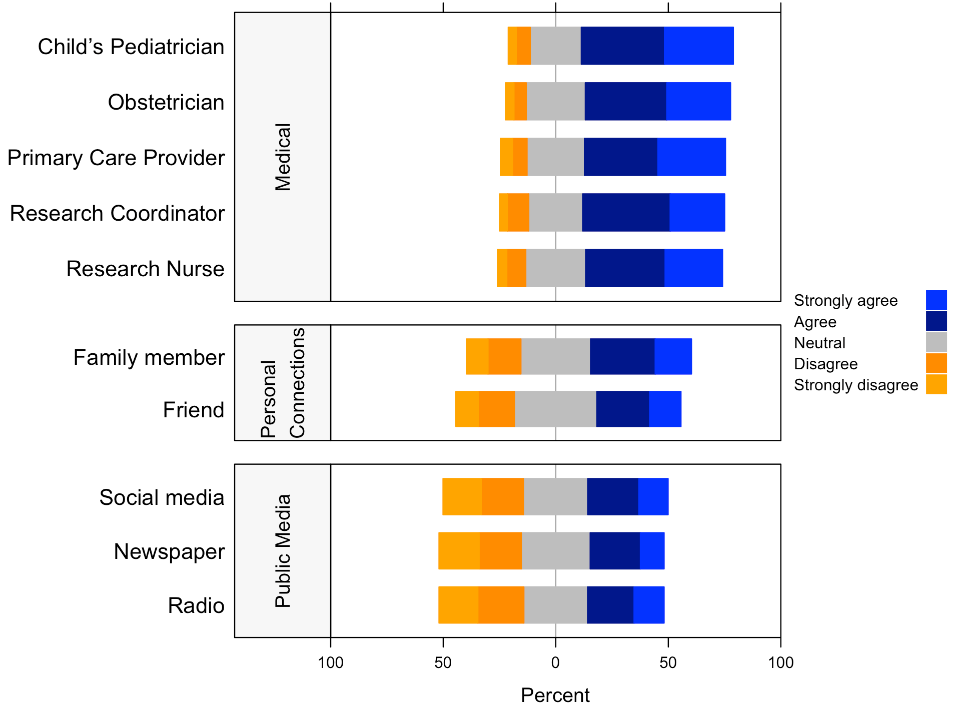

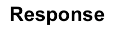

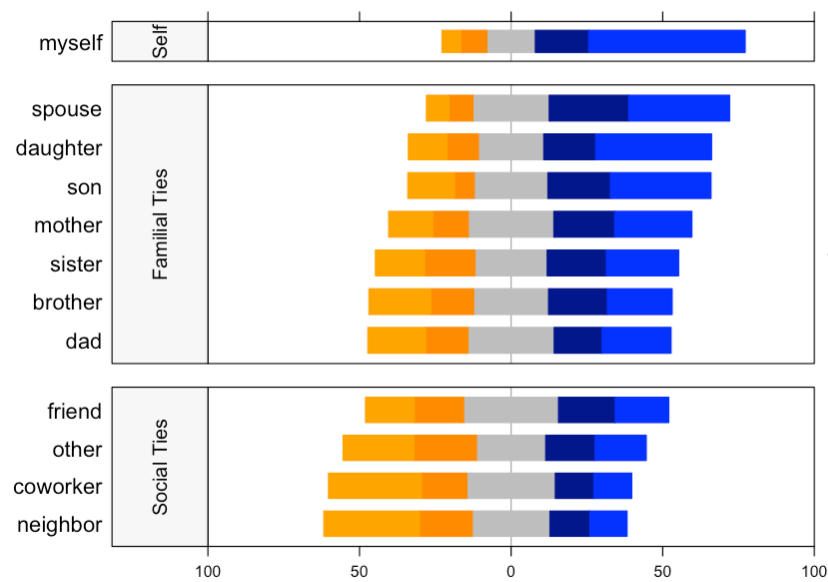


**Figure 4. Familial and social relationships that influence research participation.**  This graph shows the Likert scale of relationships that influence research. The graph visualizes the proportion of the responses reporting strongly disagree, disagree, neutral, agree, and strongly agree towards the 12 items of relationships that influence research participation. Gray stands for neutral, blue stands for positive attitude, and orange stands for negative attitude.


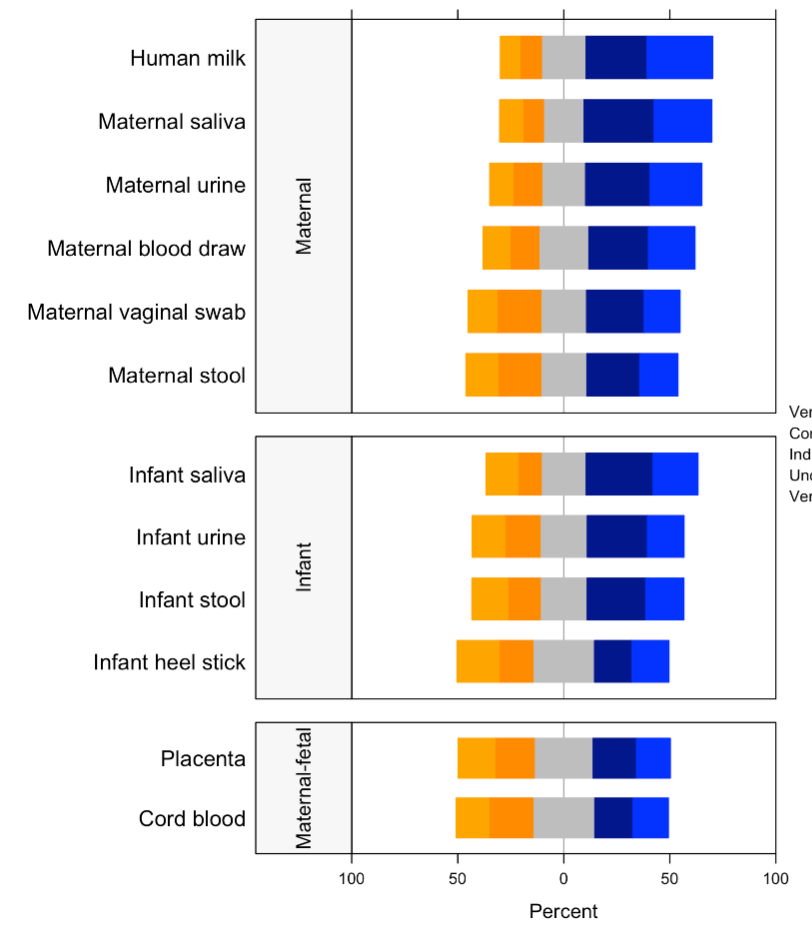

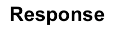

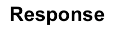

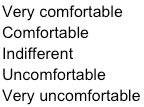

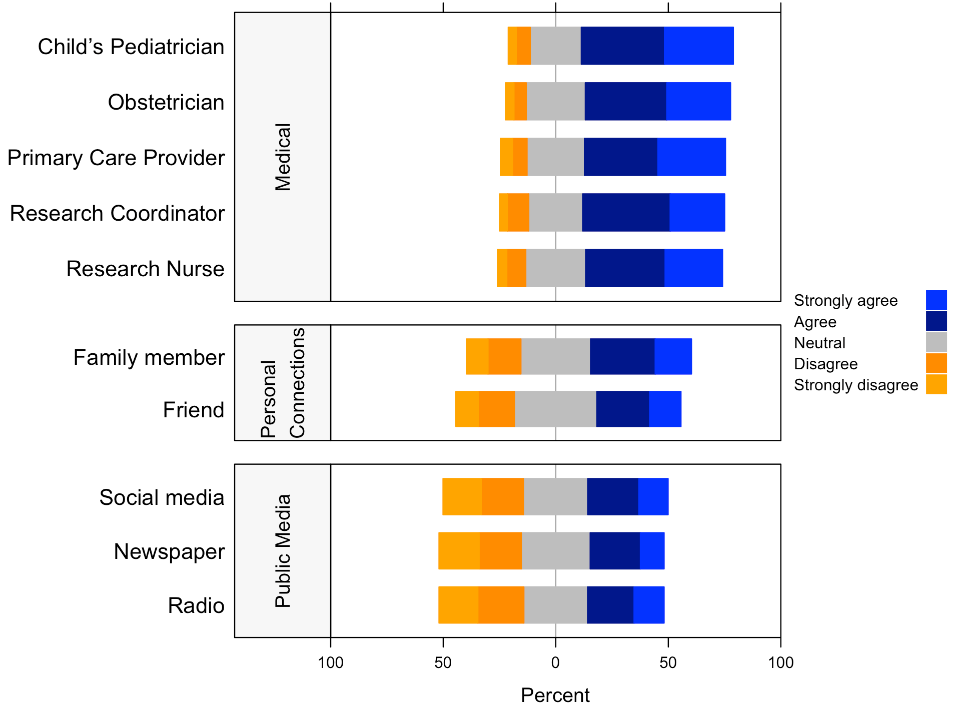

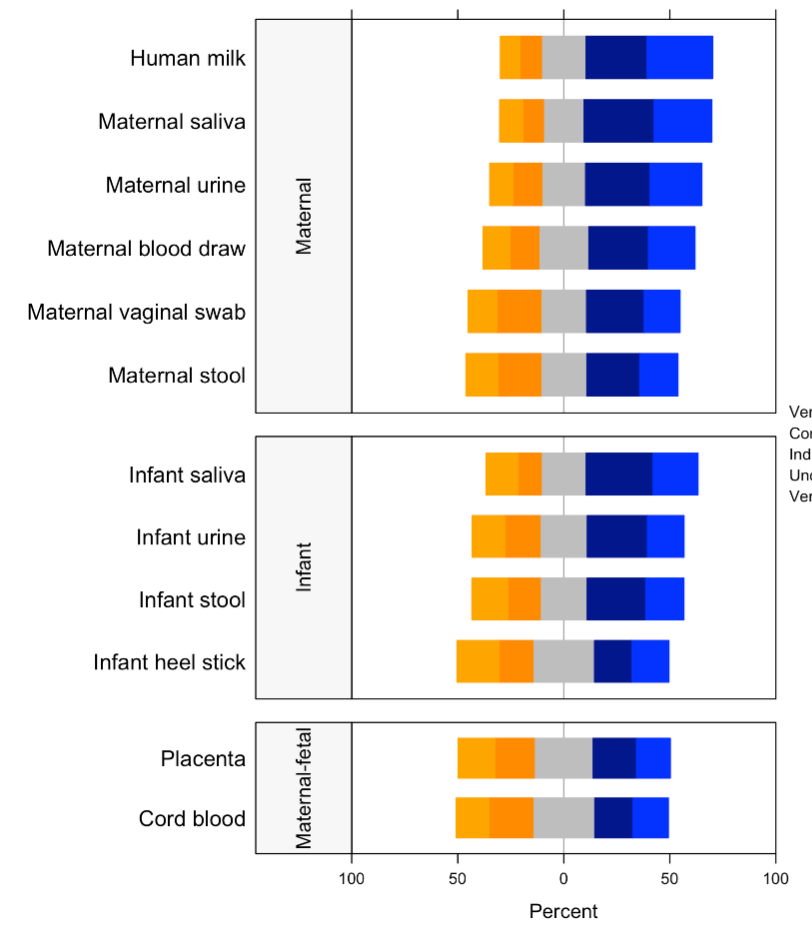

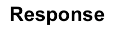

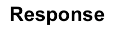

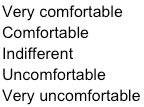


**Figure 5. Comfort providing biological samples.** This graph shows the Likert scale of comfort providing biological samples. The graph visualizes the proportion of the responses reporting very uncomfortable, uncomfortable, indifferent, comfortable, and very comfortable towards the 19 items of comfort providing biological samples. Gray stands for neutral, blue stands for positive attitude, and orange stands for negative attitude.

**Table 1: Research Participation**

| **Characteristic** | **Frequency (n)** | **Percent (%)** |
| --- | --- | --- |
| **Previously Asked to Participate in Research**  Yes  No | 185  348 | 34.7  65.3 |
| **Previous Research Participation**  Yes  No | 129  404 | 24.2  75.8 |
| **Participation in Research Related to Pregnancy^1^** |  |  |
| Yes | 74 | 60.2 |
| No | 49 | 39.8 |
| **Previous Research Topics^1^** |  |  |
| Allergy and immunology | 37 | 28.7 |
| Pain relief | 30 | 23.3 |
| Diabetes | 29 | 22.5 |
| Genetics | 25 | 19.4 |
| Nutrition and physical activity | 25 | 19.4 |
| Psychiatry and behavioral sciences | 25 | 19.4 |
| Cancer | 22 | 17.1 |
| Neurology | 22 | 17.1 |
| Obesity and weight loss | 20 | 15.5 |
| Cardiovascular health | 18 | 14.0 |
| Other | 10 | 7.8 |
| Gastrointestinal diseases | 9 | 7.0 |

^1^Only respondents who have experience of participation in research will answer these questions.

**Table 2. Digital and Social Media Preferences**

| **Characteristic** | **Frequency (n)** | **Percent (%)** |
| --- | --- | --- |
| **Preferred Form of Communication** |  |  |
| Text | 303 | 56.8 |
| Email | 296 | 55.5 |
| Call | 180 | 33.8 |
| Mail | 98 | 18.4 |
| Electronic medical record portal messaging | 48 | 9.0 |
| Other | 6 | 1.1 |
| **Preferred Time of Day** |  |  |
| Mornings | 246 | 46.2 |
| Afternoons | 224 | 42.0 |
| Lunch | 207 | 38.8 |
| Evenings | 101 | 18.9 |
| **Smart Phone Brand** |  |  |
| Apple | 367 | 68.9 |
| Samsung | 91 | 17.1 |
| LG | 43 | 8.1 |
| Other | 15 | 2.8 |
| HTC | 10 | 1.9 |
| No smart phone | 6 | 1.1 |
| Nexus | 1 | 0.2 |
| **Smart Phone Use** |  |  |
| Personal use only | 272 | 51.0 |
| Work use only | 35 | 6.6 |
| Both | 226 | 42.4 |
| **Social Media Apps** |  |  |
| Facebook | 231 | 43.3 |
| Instagram | 169 | 31.7 |
| TikTok | 49 | 9.2 |
| Snapchat | 36 | 6.8 |
| Twitter | 27 | 5.1 |
| Other | 14 | 2.6 |
| Reddit | 7 | 1.3 |
| **Use of Pregnancy-related Apps** |  |  |
| Yes | 381 | 71.5 |
| No | 152 | 28.5 |
| **Use of Breastfeeding-related Apps** |  |  |
| Yes | 242 | 45.4 |
| No | 291 | 55.6 |
