# Supplementary material for: Peripartum women’s perspectives on research study participation in the OneFlorida Clinical Research Consortium during COVID-19 pandemic
Source: J Clin Transl Sci. 2022 Oct 10;7(1):e24. doi: 10.1017/cts.2022.476 (PMC9879925; doi:10.1017/cts.2022.476)
Supplement: Supplementary file 1 [file ctssup.zip › S2059866122004769sup002.docx]

**I. Demographic Questions**

1. Do you consider Florida your primary state of residence?
   - Yes
   - No
2. Are you currently pregnant?
   - Yes
   - No
3. Which of the following categories describes your age:
   - Less than 18 years of age
   - 18-24
   - 25-34
   - 35-44
   - 45-54
   - 54+
4. Are you Hispanic or Latino?
   - No, I am not Hispanic or Latino
   - Yes, I am Hispanic or Latino
5. How would you describe yourself? (Choose one or more from the following racial groups.)
   - White (European)
   - Middle Eastern or North African
   - Black or African American
   - Asian
   - American Indian or Alaska Native
   - Native Hawaiian or Other Pacific Islander
   - Mixed race/multiple races
   - Other (please specify): _____________________
6. Please enter your current zip code: ______________
7. Are you currently breastfeeding or pumping to feed your child breast milk?
   - Yes
   - No
8. How many pregnancies have you had, including miscarriages, abortions and stillbirths? (NOT including your current pregnancy)
   - ______Pregnancies
9. What is the highest level of education you have completed?
   - 8^th^ grade or less
   - Some high school
   - High school diploma/GED
   - Some college or community college
   - Associate degree
   - Completed tech or vocational school
   - College graduate
   - Some graduate or professional school
   - Graduate or professional degree
   - I don’t know
   - Prefer not to answer
10. What is your household income?

- Under $5,000
- $5,000 to $9,999
- $10,000 to $14,999
- $15,000 to $19,999
- $20,000 to $24,999
- $25,000 to $29,999
- $30,000 to $34,999
- $35,000 to $39,999
- $40,000 to $44,999
- $45,000 to $49,999
- $50,000 to $54,999
- $55,000 to $59,999
- $60,000 to $64,999
- $65,000 to $69,999
- $70,000 to $74,999
  - more than $75,000
  - Prefer not to answer

1. What type of insurance do you have?
   - Private
   - Medicaid
   - Military-related
   - I do not have health insurance
   - Other
2. Are you WIC eligible? WIC (Woman, Infant and Children) refers to a federally funded nutrition education program for low-income pregnant, breastfeeding, and non-breastfeeding postpartum women, and to infants and children up to five years who are nutritionally at risk.
   - Yes
   - No
3. What is your current relationship status?

- Single
- In a committed relationship
- Engaged or Married
- Widowed
- Separated/Divorced

1. Do you have a smart phone?

- Yes
- No

1. What brand is your smart phone?
   - Apple
   - Samsung
   - HTC
   - LG
   - Nexus
   - Google
   - Other
2. Do you use your smart phone for work or personal use?
   - Personal
   - Work
   - Both
3. What social media platforms do you use on your smart phone? Select all that apply.
   - Facebook
   - Instagram
   - Twitter
   - TikTok
   - Snapchat
   - Other
4. Are you using or are you planning to use mobile apps related to your pregnancy?

- Yes
- No

1. Are you using or are you planning to use mobile apps related to breastfeeding your infant?
   - Yes
   - No

**II. Basic Research Knowledge**

The following questions pertain to your prior clinical research experience. The definition of clinical research is the study of human participants and their health and illness.

1. Have you ever been asked to participate in a clinical research study?
   - Yes
   - No
2. Have you ever participated in a clinical research study?
   - Yes
   - No
3. Was this clinical research study related to your pregnancy?
   - Yes
   - No
4. What topics were studied? Check all that apply.

- Nutrition & physical activity
- Obesity and weight loss
- Diabetes
- Cardiovascular health
- Neurology
- Psychiatry & behavioral sciences (Addiction, Depression, etc)
- Allergy & immunology
- Gastrointestinal diseases
- Pain relief
- Cancer
- Genetics
- Other

**III. Facilitators to Research Participation**

1. Please indicate how important each of the following people are in deciding whether you or a family member would be willing to participate in a clinical research study:

| **The importance of [family, friend or person] in participating in research is . . .** | | | | | | |
| --- | --- | --- | --- | --- | --- | --- |
|  | Strongly agree | Agree | Neutral | Disagree | Strongly disagree | N/A |
| My mother |  |  |  |  |  |  |
| My father |  |  |  |  |  |  |
| My spouse/partner |  |  |  |  |  |  |
| My brother |  |  |  |  |  |  |
| My sister |  |  |  |  |  |  |
| Myself |  |  |  |  |  |  |
| My son |  |  |  |  |  |  |
| My daughter |  |  |  |  |  |  |
| My friends |  |  |  |  |  |  |
| My neighbors |  |  |  |  |  |  |
| My co-workers |  |  |  |  |  |  |
| Other |  |  |  |  |  |  |

1. What **motivates** you to participate in a research study? Please rate the following statements based on how strongly you agree or disagree.

| **Participating in research is . . .** | | | | | |
| --- | --- | --- | --- | --- | --- |
|  | Strongly Agree | Agree | Neutral | Disagree | Strongly Disagree |
| … important to me because my own health can benefit from the findings. |  |  |  |  |  |
| … important because my husband/wife/partner values it. |  |  |  |  |  |
| … important because my friends and coworkers consider it to be a valuable activity. |  |  |  |  |  |
| …important to me because the knowledge gained from my participation can benefit someone else in the future. |  |  |  |  |  |
| … important because it can benefit the health of my community. |  |  |  |  |  |
| …largely dependent on incentives such as money and gifts. |  |  |  |  |  |
| … largely dependent on how interesting the research topic is to me. |  |  |  |  |  |
| … largely dependent on whether the study offers educational benefits and support groups. |  |  |  |  |  |
| … largely dependent on my relationship with my provider. |  |  |  |  |  |
| …largely dependent on whether childcare will be provided. |  |  |  |  |  |
| …based on how well the study is explained to me. |  |  |  |  |  |
| … based on the race of the provider/researcher conducting the study. |  |  |  |  |  |
| … based on the gender of the provider/researcher conducting the study. |  |  |  |  |  |

**IV. Barriers to Research Participation**

1. What **barriers** would prevent you from participating in a clinical research study? Please rate the following from an extreme barrier to not a barrier at all.

| **I would not participate in research because ….** | | | | | |
| --- | --- | --- | --- | --- | --- |
|  | Strongly Agree | Agree | Neutral | Disagree | Strongly Disagree |
| …I am not interested in the research topic. |  |  |  |  |  |
| …of time commitment of study visits. |  |  |  |  |  |
| …of length of study (i.e., weeks/years). |  |  |  |  |  |
| …I would need to take time off from work. |  |  |  |  |  |
| …of transportation issues. |  |  |  |  |  |
| …of possible discomforts to me such as a blood draw. |  |  |  |  |  |
| …of possible discomforts to my child such as a heel stick. |  |  |  |  |  |
| …there is no direct benefit to me. |  |  |  |  |  |
| …there is no direct benefit to my child. |  |  |  |  |  |
| ...follow-up visits are required. |  |  |  |  |  |
| ...the research goals are not well explained. |  |  |  |  |  |
| ...my family is concerned. |  |  |  |  |  |
| …my friends are concerned |  |  |  |  |  |
| ...of fear of lack of confidentiality. |  |  |  |  |  |
| ...of lack of support from medical/clinical staff. |  |  |  |  |  |
| ...of lack of interest in research from medical staff. |  |  |  |  |  |
| ...of concern it will impact my medical care from my provider |  |  |  |  |  |
| …of the possibility of receiving the placebo treatment (placebo means something with no actual effect) |  |  |  |  |  |
| … I had to collect biological samples such as stool and urine. |  |  |  |  |  |

**V. Study Preferences**

1. If I was being asked to participate in a clinical research study, I would like to receive information about the study from:

|  | Strongly Agree | Agree | Neutral | Disagree | Strongly Disagree |
| --- | --- | --- | --- | --- | --- |
| My Primary Care Provider |  |  |  |  |  |
| My Obstetrician |  |  |  |  |  |
| My child’s Pediatrician |  |  |  |  |  |
| Research Coordinator |  |  |  |  |  |
| Research Nurse |  |  |  |  |  |
| Family member |  |  |  |  |  |
| Friend |  |  |  |  |  |
| Social media |  |  |  |  |  |
| Radio |  |  |  |  |  |
| Newspaper |  |  |  |  |  |

1. In regards to receiving upcoming reminders about study appointments, what is your preferred form of communication? (Select all that apply?)
   - Text message
   - Phone call
   - Email
   - Mail
   - Electronic medical record portal messaging
   - Other
2. In regards to scheduling upcoming study appointments, what is your preferred time of day to meet for your study visits?  (Select all that apply)
   - Mornings
   - Lunch Time
   - Afternoons
   - Evenings
3. How willing would you be to release your personal health information for research purposes if assured that your personal health information will be kept private/confidential?

- Very Likely
- Somewhat Likely
- Neutral
- Somewhat unlikely
- Strongly unlikely

1. How likely would you participate in a clinical research study that involved being randomized (equal chance of being placed in a treatment group or placebo [no treatment] group)?

- Very Likely
- Somewhat Likely
- Neutral
- Somewhat unlikely
- Strongly unlikely

1. How comfortable do you feel with providing the following biological samples for you and your infant as part of a clinical research study?

|  | Very Comfortable | Comfortable | Indifferent | Uncomfortable | Very uncomfortable |
| --- | --- | --- | --- | --- | --- |
| Human milk |  |  |  |  |  |
| Maternal stool |  |  |  |  |  |
| Maternal urine |  |  |  |  |  |
| Maternal vaginal swab |  |  |  |  |  |
| Maternal saliva |  |  |  |  |  |
| Maternal blood draw |  |  |  |  |  |
| Cord blood |  |  |  |  |  |
| Placenta |  |  |  |  |  |
| Infant stool |  |  |  |  |  |
| Infant urine |  |  |  |  |  |
| Infant saliva |  |  |  |  |  |
| Infant heel stick |  |  |  |  |  |
